# Movement Economy in Soccer: Current Data and Limitations

**DOI:** 10.3390/sports6040124

**Published:** 2018-10-23

**Authors:** Filippo Dolci, Nicolas H. Hart, Andrew Kilding, Paola Chivers, Ben Piggott, Tania Spiteri

**Affiliations:** 1 School of Health Science, University of Notre Dame, Fremantle, WA 6160, Australia; benjamin.piggott@nd.edu.au; 2 School of Medical and Health Science, Edith Cowan University, Perth, WA 6027, Australia; n.hart@ecu.edu.au (N.H.H.); t.spiteri@ecu.edu.au (T.S.); 3 Institute for Health Research, University of Notre Dame, Fremantle, WA 6160, Australia; paola.chivers@nd.edu.au; 4 Exercise Medicine Research Institute, Edith Cowan University, Perth, WA 6027, Australia; 5 Sports Performance Research Institute New Zealand, AUT University, Auckland 1010, New Zealand; andrew.kilding@aut.ac.nz

**Keywords:** movement economy, running economy, aerobic energy cost, soccer, endurance, aerobic fitness

## Abstract

Soccer is an intermittent team-sport, where performance is determined by a myriad of psychological, technical, tactical, and physical factors. Among the physical factors, endurance appears to play a key role into counteracting the fatigue-related reduction in running performance observed during soccer matches. One physiological determinant of endurance is movement economy, which represents the aerobic energy cost to exercise at a given submaximal velocity. While the role of movement economy has been extensively examined in endurance athletes, it has received little attention in soccer players, but may be an important factor, given the prolonged demands of match play. For this reason, the current review discusses the nature, impact, and trainability of movement economy specific to soccer players. A summary of current knowledge and limitations of movement economy in soccer is provided, with an insight into future research directions, to make this important parameter more valuable when assessing and training soccer players’ running performance.

## 1. Introduction

Soccer is a multi-skilled sport requiring a combination of physical, technical, and tactical abilities to produce a superior performance [1,2,3]. Soccer players must perform various multifaceted intermittent activities at different intensities, such as sprints, changes of direction, jumps, tackles, and skill-based activities, with the ball, over 90 min of play, in a dynamic and unpredictable environment [1,4]. Physiologically, the execution of these activities throughout a game requires a complex interaction of both anaerobic and aerobic energy systems. Indirect estimation of oxygen consumption suggests that the aerobic pathway dominates energy delivery by providing approximately 90% of the total metabolic cost of a match [2,5]. This high oxygen taxation results from the aerobic energy system’s contribution to performing continuous submaximal activities (mainly reliant on oxidative metabolism); repeated high-intensity activities (such as sprinting, for instance), where oxygen may contribute up to 40% of the energy required [6]; and re-establishing metabolic processes and anaerobic stores during recovery periods [7]. Hence, aerobic fitness appears to be crucial for athletes to physically compete in the intermittent nature of soccer matches. However, while the focus is often given to the maximal aerobic capacity of soccer players [8], there are other aerobic parameters that may influence running outputs during games. Movement economy (ME) is a key parameter of aerobic fitness, determined by various combined physiological and biomechanical characteristics of an athlete [9]. Movement economy has been investigated extensively in endurance athletes, such as runners, cyclists, and triathletes [9,10] indicating a strong ability to predict athletic performance [11,12]. Conversely, consideration of ME in soccer players, despite the prolonged duration of a soccer game, is much scarcer [13,14,15,16,17,18,19], and has yet to be reviewed. Within the current literature on ME in soccer players [13,14,15,16,17,18,19], there is limited data, along with ongoing debates regarding the poor specificity of common ME assessment to team-sports [14,20]. This article aims to review and summarize the current scientific data of ME in soccer players, while also providing a critique of current methods of assessment and data produced to date, in order to identify ways in which use of ME can be advanced in soccer contexts.

## 2. Methods

The following search terms were used in this literature review: running economy, soccer, aerobic fitness, endurance, football, training, and energetic cost. The search was undertaken using Google scholar and eResources, accessed through The University of Notre Dame Australia’s online library. All peer-reviewed articles presenting movement economy (running economy) data in soccer players have only been included in the review analysis (articles where data of soccer players were averaged with other participants excluded). In addition, articles discussing statistical changes in movement economy, but not reporting player values, have been included. No specific age, gender, level, or period and method of economy assessment have been considered as factors for inclusion or exclusion criteria.

## 3. Definition and Concept of Movement Economy in Soccer

Movement economy is generally defined as the aerobic energy cost required to perform a submaximal task, providing information about an athlete’s aerobic efficiency while exercising at submaximal velocities [9]. In soccer players, similarly to endurance runners, ME has been commonly measured and considered as the submaximal aerobic efficiency when running in-line at constant speed on a treadmill. For this reason, both in long-distance runners and soccer players, ME has been more commonly referred to as running economy [9]. Nonetheless, ME is specific to the task, and might vary when performing different activities and in different environments [20]. Hence, considering ME as the ability to run efficiently at a constant submaximal speed, and in a straight line only, might be a reductionist approach in soccer, and may reflect only a partial picture of a soccer player’s ME, given the plethora of directional changes present in the act of training or competition. A more appropriate and complete concept of ME in soccer should allude to the submaximal aerobic efficiency of soccer players when performing multiple sport-related activities under context-specific situations. This concept is supported throughout this review.

## 4. Determinants of Movement Economy in Soccer

**Figure 1:**
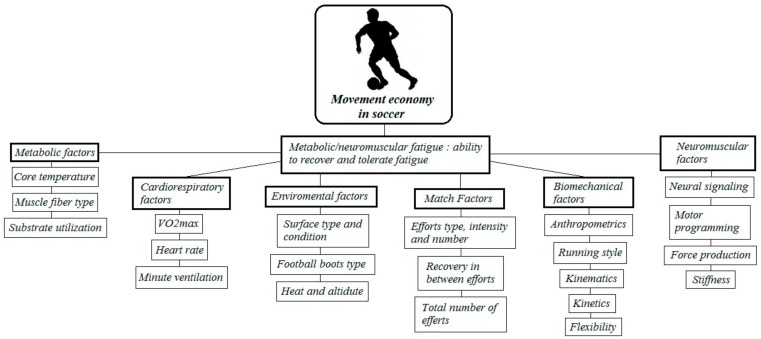
Factors affecting movement economy in soccer, adapted from Barnes and Kilding [9].

During soccer, running performance has been often reported to decrease over the match [4], hence suggesting that the ability to sustain long exercise activities (endurance) is a crucial aspect to preserve optimal physical performance. Movement economy is a well-established determinant of endurance, which represents the complex resultant of an athlete’s metabolic, cardiorespiratory, biomechanical, and neuromuscular efficiency [9,21]. Metabolic efficiency refers to the ability of an athlete to utilise available energy, which can be affected by core temperature, muscle fibre type, and substrate utilisation [9]. Cardiorespiratory efficiency refers to the work output required to deliver and utilise oxygen in the working muscle, and is related to heart rate, minute ventilation, and VO_2max_. Biomechanical and neuromuscular efficiency represents the ability of the musculoskeletal and neural systems to translate power output into effective movement output. Specifically, musculoskeletal efficiency is influenced by factors such as anthropometrics, movement style, kinetics, kinematics, and flexibility, while neuromuscular efficiency is influenced by the speed of neural transmission, motor programming, force production, and stiffness [9]. Since fatigue can affect ME [22], and the high-intensity periods of soccer playing have been often observed to induce both a transient and prolonged effect of fatigue during a match [23], it is rational to expect metabolic, cardiorespiratory, biomechanical, and neuromuscular efficiency fluctuations according to players’ fatigue status. This players’ fatigue status is intuitively affected by the nature of the game and the players’ ability to recover fast/tolerate fatigue; it can be the latter key determining factor affecting players ME during a game (Figure 1). 

Specifically, when considering metabolic efficiency, there is an advantage in oxidating a higher percentage of carbohydrate vs fats, because of the greater energy produced by the former substrate per oxygen mole [24]. However, the decrease in muscle glycogen concentrations or the increase in catecholamine concentrations, induced by soccer activity, may increase lipid turnover throughout a game [25] and, in turn, negatively affect metabolic efficiency. In addition, cardiorespiratory efficiency, low heart rate, and minute ventilation have been constantly reported as an economical advantage, because they reflect lower respiration, which can represent up to 6–7% of the total oxygen cost of exercise [9,26]. The increase in ventilation following high-intensity soccer activities can, therefore, also compromise cardiorespiratory efficiency. Neuromuscular efficiency could also be impaired by high-intensity periods of soccer games. Specifically, efficient neural signalling, along with the ability to produce high power and lower body stiffness, have all been proposed as advantageous factors for neuromuscular efficiency [9], because allowing for optimal motor unit recruitment, greater running propulsion, and elastic energetic reutilization, respectively, can translate into a lower magnitude and number of muscular contraction (i.e., energetically costly to move [9]). However, these neuromuscular factors have been reported to be negatively affected by central and peripheral fatigue induced by high-intensity activities [27,28,29]. Lastly, there is evidence that neuromuscular fatiguing activities, as occurring during soccer matches, alter an athlete’s kinematic characteristics [30,31]. Since high-performing athletes initially tend to adopt the most economical kinematic features for a given movement [9], a change in this might imply that they cannot preserve their optimal biomechanical efficiency following soccer-related activities. Therefore, in soccer players, it might be particularly important to consider ME changes over a match or soccer-specific high-intensity activities, and evaluate strategies to mitigate ME fluctuation, in order to preserve optimal metabolic, cardiorespiratory, and neuromechanical efficiency.

## 5. Current Movement Economy Testing Approaches in Soccer

In soccer players, ME has been only measured in laboratory conditions when running at submaximal speeds on a treadmill, for time periods long enough to reach a physiological steady state [9]. Such an approach has been used because it is valid and practical to use [9] but, also, because a more specific and validated ME test for soccer has been lacking. Other standardized and reliable protocols to measure ME, specifically in team-sports, have been only recently been proposed [20], and still require validation. The speeds used for assessing ME while running, in soccer players, have ranged from 7 km/h to 14 km/h [13,16,18,32,33,34,35], or corresponded to speed associated with LT_an_ [14,15], where running intervals have not been longer than 5 min [13,14,15,16,18]. Physiological steady state is typically verified by an athlete maintaining blood lactate concentration similar to pre-activity baseline (within 1 mmol/L) [36], or a respiratory exchange ratio (RER) of <1.0 [11]. To provide a measure of ME, studies analysing different athletic populations have generally expressed the mean VO_2_ obtained during the final minutes or so of running at the prescribed steady state velocity, either in relative terms per ratio of body mass^−1^ per min (or per km), or with an allometric scaling to the power of 0.67 or 0.75 (e.g., mL/kg^0.67^/min or mL/kg^0.75^/min) [37,38]. Nonetheless, different exponents can be used when comparing ME between subjects with different body mass. For instance, specifically to soccer, allometric scaling, raising body mass to the power of 0.60, has been applied to better express ME between players of young (14 years) and adult (24 years) ages [16]. Tests employed to assess soccer players’ ME and relative normative values, are presented in Table 1 and Table 2.

Expressing ME as a relative or allometrically scaled oxygen cost has been the most commonly applied method to characterise soccer players [13,14,15,16,18,19,33,34,35,39,40,41], however, it is noted that recent studies have also suggested considering ME in relation to substrate utilisation [24,42]. Carbohydrates have a greater energy equivalent per oxygen mole than fats and, consequently, a measure of oxygen consumption alone can fail to account for the real energy produced by aerobic metabolism when athletes rely on different proportions of substrates during activities. Therefore, considering ME as the gross caloric unit cost (kcal/kg/km), extrapolated by values of gas exchange via the updated non-protein respiratory quotient equations [43], has been recently recommended [24,42], and may provide greater insight into ME qualities of athletes, including soccer players.

## 6. Limitations of Current Movement Economy Assessments in Soccer

Current ME tests, applied in soccer, evaluate a player’s aerobic energy cost while running in-line at a constant submaximal speed, usually in laboratory conditions [9]. Hence, the nature of current ME assessment poses limitations into fully capturing a player’s aerobic efficiency when performing various on-field soccer-specific activities (i.e., what is the transfer of ME measures from lab to pitch?). Indeed, there are several factors that could influence the applicability of ME measures assessed in the lab to match-specific soccer running performance. Firstly, the interaction of surface and footwear used during laboratory assessments are different from those during regular soccer practice/matches, thus, environmental validity is a limitation. In fact, these variables have been shown to have an impact on ME [44,45] and, therefore, ME when moving over a grass pitch and wearing soccer boots might differ from ME assessed on treadmill or indoor surfaces with different types of footwear. Furthermore, ME is commonly measured during constant in-line running and, therefore, might not fully account for the multidirectional intermittent movement performed by soccer players, as aerobic movement efficiency might rely on different neuromechanical and physiological factors. For instance, from a neuromechanical prospective, greater muscular activity [46] and lower tendon stiffness result in less elastic energy reutilisation [47] when accelerating, decelerating, and changing direction continuously, compared to running in-line at a constant speed. Further, metabolic responses when performing soccer specific activities, such as runs with change of directions, have been reported to differ from in-line running, as indicated by the greater oxygen consumption and lactate production over a wide range of activities at submaximal intensities [20,48]. In addition, soccer activity is interspersed by high-intensity intermittent activities [3], which may acutely impair determinants of ME. Hence, current ME assessments may not be sensitive enough to provide a relevant index of players’ ME over large periods of a game. This illustrates the limitations of current ME investigations in soccer, which have yet to investigate ME over more soccer-specific environment/activities.

## 7. Impact of Movement Economy on Soccer Performance

The poor specificity of ME assessment and values produced often lead to debates regarding the impact of ME on team-sports running performance [20]. Hence, ME in soccer has been commonly underlooked. Nonetheless, during soccer, large parts of the game are played at submaximal speeds, suggesting that players showing high movement efficiency at given speeds might have a relevant advantage for the running performance over a 90 min game. Specifically, from indirect values of oxygen consumption [5,28] and time spent playing at different speed thresholds [4], the greatest proportion of matches are played at speeds below the velocity associated with VO_2max_ (vVO_2max_). Moreover, it has been reported that approximately 40–45 min of matches are played below intensities commensurate with LT_an_ [49]. Hence, if appropriately considering ME as the aerobic energy cost required to perform various submaximal game-specific tasks, it is intuitive that players with a better ME may take advantage by using less energy to execute most of the movements required throughout a game and, thus, preserve valuable energy for important high-intensity activities. This could be particularly beneficial to mitigate the fatigue-related drop in physical performance, often observed as a progressive drop in total and high-intensity running during a 90 min game [4,23,50,51,52]. Moreover, players with better ME might spend less overall energy for the same work over a game and, in turn, require shorter post-match recovery periods to re-establish baseline energetic resources. Recent research has also produced indirect data regarding the potential beneficial role of ME during high-intensity intermittent activities, showing that vVO_2max_, which is an index of ME and VO_2max_ together, correlated to repeat sprint ability (RSA) performance (decrement), better than VO_2max_ alone [53,54]. While the underlying reasons for this needs to be further investigated, it is hypothesised that ME was the main contributor underpinning the correlation between vVO_2max_ and RSA. Hence, mechanisms contributing to ME may play a relevant role over the performance of maximal repeated activities too, which are crucial in soccer [55]. This further supports the importance of considering ME and related determinants to evaluate and improve soccer players’ running performance over a multitude of game-specific activities. 

## 8. Normative Data in Soccer Players

Few studies have provided measures of ME in soccer players in an attempt to investigate the discriminant ability of this parameter [16,18,19,22,28] or evaluate changes following training interventions [13,14,15,17,26]. Nonetheless, because of the reductionist approach adopted, which consisted of evaluating ME only while running in laboratory conditions, current values of ME might not be fully representative of players’ soccer-specific ME. In addition, comparisons of current ME values across different studies are limited by (1) heterogeneous samples, (2) variations in protocol speed, duration, and (3) expression of ME. Also, (4) ME has been shown to be sensitive to training [13,14,15,17,27], thus, the different time periods of assessment (e.g., pre-season, competitive season) among studies complicate their comparability. Table 1 and Table 2 report data of current ME for soccer players from existing cross-sectional and training studies, respectively. 

The majority of ME studies in soccer have applied different allometric scaling methods, making it difficult to establish normative data for soccer players. Despite this, it has been reported that elite soccer athletes (first and second league) exhibit better ME compared to third league players, despite similar VO_2max_ [18] suggesting that ME, even when assessed using treadmill in-line running, could be an important differentiator in soccer players. Furthermore, a study comparing ME between young (~14 years) and adult (~24 years) players indicated significantly better ME in adults, but when ME values were scaled to body mass, 0.60, differences were non-existent [16]. This could be due to tests occurring at the same fixed speed (7 km/h) for both groups, which likely corresponded to a higher relative speed for the younger group. Higher relative running intensity increases the oxidation of CHO compared to fat, and might have resulted in similar oxygen uptake between young and adult players, regardless of different energy production via substrate oxidation (CHO produces more energy per O_2_ mole than fats) [29,30]. An analysis of ME by considering substrate utilisation might have been more appropriate in this study. When comparisons of ME between early and late maturing soccer players (defined by average skeletal age of 12 and 16 years, respectively) were performed, Segers et al. [28] did not report differences in ME due to physical maturity levels, however, there was a trend towards better ME for the older group. The small sample size of this study (12 players in total) may have been underpowered to detect statistically significant differences. While the limited and different ME tests make it difficult to establish normative or representative ME data for soccer players, from the few studies presented in the literature, it appears that higher-level players could exhibit better ME during in-line running at constant speed. However, whether values of this parameter differ across players of different ages remain unclear, and whether it varies between different positions or gender, as with other parameters of aerobic fitness (i.e., VO_2max_), still needs investigation. In addition and, possibly, more importantly, whether ME assessment over more soccer-specific movements could produce different and more contrasting scores between players of differing levels and ages, is still unknown. Last but not least, ME has been constantly observed to decrease over endurance events because of fatigue processes [40]. Since the effect of fatigue is likely to be more rapid and intermittent in soccer matches where unpredictable and demanding high-intensity movements occur since the first minutes of a game, values of ME decrement/fluctuation during, and after, fatiguing activities might be an additional valuable normative data to consider, in order to compare ME abilities across different soccer players.

## 9. Trainability of Movement Economy in Soccer Players

Despite various studies to improve ME in endurance runners [10], only a few have assessed the impact of training interventions on soccer players’ ME (see Table 2), with all of these studies measuring ME during in-line running in laboratory conditions. It is still difficult to make a direct comparison between studies and draw definitive conclusions regarding the effectiveness of specific types of training on ME, because of the different testing protocols, players level/gender, and period of intervention, however, it has been reported that ME can be improved, from 0.71 ± 0.29% to 13 ± 0.4.77% following training interventions.

One study in female soccer players investigated the impact of combined strength and plyometric training on ME for 11 weeks over the off-season period, reporting that there was no significant change as a result of the intervention. However, the lack of a control group does not allow investigation as to whether such an intervention still had a positive impact in preserving ME over the off-season period, where the absence of regular soccer activity might potentially result in performance decrement [56]. Other studies have focused on the effect of high-intensity interval training (HIIT) or concurrent HIIT and strength training on elite male soccer players only (see Table 2). These studies have constantly reported ME improvements ranging from +2.53% to +13.4%. Helgerud, Engen [14], using elite junior soccer players, observed a significant improvement in ME (+7.6%) after implementation of an 8 week HIIT program (4 × 4 min running at ~90% maximum heart rate) performed in-season, twice per week, with no significant difference in the control group. During the pre-season, Impellizzeri, Marcora [15] also observed a similar beneficial effect by using the same HIIT protocol to train under-20 elite players, either by adopting in-line running intervals or sport-specific small-sided games at the same intensity, indicating that different exercise modes can impact on ME in soccer players. Larger significant improvements (+10%) were also reported in-season, in under-14 elite soccer players, when performing a HIIT protocol, running on a soccer-specific circuit [40]. Here, the higher improvement could be due to the lower initial level of fitness which can be expected in younger players participating in the study. However, such changes cannot be attributed solely to the HIIT intervention in this study, due to the lack of a control group. Similarly, Helgerud, Rodas [13] reported a significant improvement in ME in elite soccer players after an 8 week pre-season program involving 4 × 4 min of HIIT at ~90% maximum heart rate, concurrently with maximal lower-body strength training. While HIIT performed with strength training led to smaller ME changes (+3.5%) in this study, comparative to previous studies implementing HIIT only, it is difficult to draw comparisons. Specifically, the study by [13] did not use a control group either and, therefore, it is unknown if changes were due to the training intervention only, and whether the smaller improvements in ME, compared to other studies implementing HIIT in soccer practice, were simply due to the higher level of players used in the study, or if concurrent training attenuated improvements in ME. Fastest improvements in ME have been observed in elite players following a short HIIT microcycle (10 sessions over two weeks) conducted at the end of the regular season with no changes in control group. However, in this study, the control group did not perform any additional training, hence, it is unclear if improvements observed in ME for the intervention group were induced by the specific HIIT programme, or simply due to the additional volume of activity performed. 

Overall, in soccer players, the implementation of HIIT with long intervals (4 min) and HIIT with strength training along with soccer-specific practice, can significantly and rapidly (<8 weeks) improve ME while running in-line at constant speed, regardless of the period of the season (off-, pre-, in-season). High-intensity interval training has the potential to optimize economy-related factors, such as mitochondrial efficiency, cardiac output, force production, and antagonist co-activation [57,58]. However, in soccer, a limitation of studies is that there was no a control group for comparison [13,15,32,35,39,40], and only one study matched the additional volume constituted by HIIT in the experimental group with extra activity volume in the control group [14]. Therefore, clear evidence that the HIIT mechanisms alone are responsible for ME improvements in soccer players is still required. Furthermore, studies involving HIIT interventions with control groups performing similar total training volume, is warranted. Such study designs would provide a higher level of evidence regarding the mechanism of ME improvements following HIIT implementation. 

Last but not least, assessing effects of training intervention on ME when performing soccer-specific tasks, rather than in-line running only, and the ability to preserve optimal ME following high-intensity periods, might be more relevant to understanding the value of implementing these approaches to improve aerobic efficiency under context-specific situations.

## 10. Conclusions

Movement economy is a relevant parameter of aerobic fitness during long and exhaustive sports, such as soccer. In soccer players, ME has been only assessed when running in-line and at constant speed in laboratory conditions. Measures produced by this approach are able to discriminate players based on playing standard, and tests have been sensitive to specific training interventions, such as HIIT. Nonetheless, the current ME testing methodology has limitations with regards to transferability to ME during soccer games. Indeed, the multilateral and intermittent activities that soccer players have to perform over a 90 min game alter physiological and neuromechanical aspects of movement, and can determine a different and more specific ME. Also, the various high-intensity soccer activities, which are critical and recurrent in soccer, can have a unique impact on ME, which can significantly affect players’ economy over the entire match. A further limitation is that a player’s ability to preserve optimal ME during a game cannot be assessed by current ME testing methodology. Therefore, it is important to highlight that current ME tests in soccer cannot provide a complete profile of soccer players’ efficiency when performing game-specific activities throughout the entire game and, furthermore, the validity of ME data presented in the literature is questionable. To further advance our understanding of the relevance and trainability of ME in soccer players, more consistent and specific approaches to assess ME under multiple soccer-specific tasks are required. 

## Figures and Tables

**Table 1:** Studies assessing movement economy in soccer players without a training intervention.

Study	Player Characteristics (Age)	ME Protocol	Period of Baseline Test	Baseline ME (Protocol Speed)
Chamari, et al. [16]	21 young male soccer players (14 ± 0.4 years)	Running on a treadmill with 5.5% inclination for 4 min at 7 km/h	Second half of the season	39.2 ± 2.9 mL/lbm/min (7 km/h) 0.34 ± 0.02 mL/lbm/m (7 km/h) 1.65 ± 0.04 mL/lbm^−0.60^/m (7 km/h)
	24 adult elite soccer players from Tunisian national team (24 ± 2 years)			36.0 ± 3.1 ** mL/lbm/min (7 km/h) 0.30 ± 0.02 ** mL/lbm/m (7 km/h) 1.65 ± 0.08 mL/lbm^−0.60^/m (7 km/h)
Ziogas, et al. [18]	53 Professional soccer players from Greek division A (26.2 ± 4.9 years)	Running on a treadmill with 3% inclination. After the initial speed of 10 km/h was increased by 2 km/h every 3 min until volitional exhaustion. ME calculated at 12 km/h.	Early pre-season	44.6 ± 2.9 mL/kg/min (12 km/h)
	46 Professional soccer players from Greek division B (25.9 ± 5.2 years)			44.4 ± 2.8 mL/kg/min (12 km/h)
	30 Semi-professional soccer players from Greek division C (25.6 ± 4.5 years)			46.4 ± 3.9 ** mL/kg/min (12 km/h)
Segers, et al. [41]	6 early mature soccer boys Skeletal age = 16.0 ± 1.1 years (13.2 ± 1.0 years)	Running on treadmill with 1% inclination for three 6 min interval at 8, 9.5 and 11 km/h. Rest 5 min in between. ME calculated for each interval.	Not reported	32.6 ± 3.0 mL/kg^0.98^/min (8 km/h) 35.3 ± 3.3 mL/kg^0.98^/min (9.5 km/h) 39.3 ± 3.3 mL/kg^0.98^/min (11 km/h)
	7 late matures soccer boys Skeletal age = 14.3 ± 0.6 years (14.4 ± 0.5 years)			30.0 ± 4.4 mL/kg^0.98^/min (8 km/h) 32.3 ± 5.6 mL/kg^0.98^/min (9.5 km/h) 35.1 ± 5.7 mL/kg^0.98^/min (11 km/h)
Hoppe et al. [34]	11 professional soccer players from 3rd level in Germany (23.8 ± 3.0 years)	Running on a treadmill with 1% inclination for 4 min at 10 km/h.	Pre-season	2.8 ± 0.2 L/min (10 km/h) 36.5 ± 1.8 mL/kg/min (10 km/h) 107.8 ± 3.9 mL/kg^−75^/min (10 km/h)
Nilsson and Cardinale [19]	23 elite male soccer players from the top Swedish league with higher aerobic Power: VO_2max_ = 59.7 ± 2.3 mL/kg/min (22.5 ± 3.3 years)	Running on a treadmill at 0% gradient, 4 min bouts at 10, 12, 14, and 16 km/h. Between the run at each speed level the participants had one min of rest when a blood sample was collected.	End of season	~39.0 mL/kg/min (12 km/h) ~43.5 mL/kg/min (14 km/h) ~49.5 mL/kg/min (16 km/h)
		ME calculated for every interval but not reported for the first one (10 km/h).		
	17 elite male soccer players from the top Swedish league with lower aerobic power: VO_2max_ = 53.2 ± 2.0 mL/kg/min (26.8 ± 4.8 years)			~37.5 mL/kg/min (12 km/h) ~42.0 mL/kg/min (14 km/h) ~47.5 mL/kg/min (16 km/h)
McCormack, et al. [33]	10 National Collegiate Athletic Association Division I women soccer players (19.5 ± 1.0 years)	Running on a treadmill (gradient not reported) for 4 min at 12.0 km/h.	Off-season	39.8 ± 1.8 mL/kg/min (12 km/h)

Notes: HR_max_: maximum heart rate; ME: movement economy; vLT_an_: velocity at the anaerobic lactate threshold; lbm: lean body mass; * different between pre- and post-intervention; ** different between study groups.

**Table 2:** Studies assessing movement economy in soccer players with a training intervention.

Study	Player Characteristics (age)	Testing Protocol	Period of Baseline Test	Pre-Intervention ME (Protocol Speed)	Intervention	Post-Intervention ME (Protocol Speed)
Impellizzeri, et al. [15]	20 male elite soccer players (17.2 ± 0.8 years)	Running on treadmill with 3% inclination for 10 min at 9 km/h then speed increased incrementally 1 km/h every 5 min. ME was calculated at the vLT_an_.	4 weeks before in-season	0.73 ± 0.03 mL/kg^0.75^/m (vLT_an_ = 11.3 ± 0.7 km/h)	HIIT: 4 × 4 min SSGs at 90–95% of HR_max_ with 3 min active rest periods. Twice a week × 8 weeks along with regular soccer practice	0.71 ± 0.03 mL/kg^0.75^/m (vLT_an_ 12.4 ± 0.5 km/h) 2.74 ± 0%↑
	20 male elite soccer players (17.2 ± 0.8 years)			0.72 ± 0.03 mL/kg^0.75^/m (vLT_an_ = 11.2 ± 0.6)	HIIT: 4 × 4 min continuous running at 90–95% of HR_max_ with 3 min active rest periods. Twice a week × 8 weeks along with regular soccer practice	0.70 ± 0.04 mL/kg^0.75^/m (vLT_an_ 12.2 ± 0.4) 2.78 ± 0.33%↑
Chamari, et al. [40]	18 male national level soccer players (14 ± 0.4 years)	Running on a treadmill with 5.5% inclination for 4 min at 7 km/h.	Just after mid-season	38.8 ± 2.1 mL/kg/min (7 km/h)	HIIT: 4 × 4 min HIIT on the Hoff track, separated by 3 min of active recovery. Twice a week × 8 weeks along with regular soccer practice.	33.6 ± 2.2 * mL/kg/min (7 km/h) 13. ± 0.4.77%↑
				0.90 ± 0.04 mL/kg^0.75^/m (7 km/h)		0.81 ± 0.05 * mL/kg^0.75^/m (7 km/h) 10 ± 0.25%↑
Helgerud, et al. [14]	9 male junior elite soccer players (18.1± 0.8 years)	Running on a treadmill with 3% inclination for 5 min stages starting. Starting speed at intensity corresponding to 60% VO_2max_, and then increased by 1 km/h at every stage (after 20 s recovery for blood samples). ME was calculated at the interval intensity corresponding to LT.	Beginning of the season	0.75 ± 0.05 mL/kg^0.75^/m (vLT_an_ = 11.1 ± 0.7)	HIIT: 4 × 4 min continuous running at 90–95% of HR_max_, with 3 min active recovery jogging at 50–60% of HR_max_, Twice a week × 8 weeks along with soccer practice	0.70 ± 0.04 * mL/kg^0.75^/m (vLT_an_ = 13.5 ± 0.4) 6.67 ± 0.2%↑
	10 male junior elite soccer players (18.1 ± 0.8 years)			0.75 ± 0.04 mL/kg^0.75^/m (vLT_an_ = 11.7 ± 0.4)	Extra technical training, such as heading drills, practicing free kicks, and exercises related to receiving the ball and changing direction	0.74 ± 0.04 mL/kg^0.75^/m (vLT_an_ = 11.5 ± 0.2) 1.33 ± 0%↑
Helgerud, et al. [13]/Helgerud, et al. [39]	21 male elite soccer players (25 ± 2.9 years)	Running on a treadmill with 5.5% inclination at initial speed corresponding to an intensity of 50–60% VO_2max_, then increased to 11 km/h for 5 min. ME was calculated during this last 5 min.	First pre-season	0.85 ± 0.3 mL/kg^0.75^/m (11 km/h)	HIIT+ strength: 4 × 4 min running on a treadmill (5.5% inclination) at 90–95% of HR_max_ with 3min active rest periods jogging at 50–60% of HR_max_. After a 15 min break, 4-repetition maximum of half-squats in 4 series. If 5 repetitions were managed the load was increased. 3 min rest between series. Twice per week per 8 weeks	0.82 ± 0.3 * mL/kg^0.75^/m (11 km/h) 3.53 ± 0%↑
Christensen, et al. [17]	7 male elite soccer players (26.8 ± 4.8 years)	6 runs over 2 different days. Bouts of 4 min Each bout consisted of 1 min of rest standing on the treadmill followed by 4 min of running at 75% MAS (average speed = 14.1 km·h^−1^) were calculated during the final 30 s of each 4 min running bout	Off-season just after last match	197.8 ± 10.2 mL/kg/km (MAS average = 14.1 km/h)	HIIT: 10 training sessions mainly consisting of aerobic high-intensity training (8 × 2 min at 87.7% ± 1.2% with 1 min recovery in between) and speed endurance training (10–12 × 30 s all-out sprints with rest of similar duration) performed over two weeks.	192.8 ± 7.2 * mL/kg/km (MAS average = 14.1 km/h) 2.53 ± 0.29%↑
	11 male elite soccer players (26.8 ± 4.8 years)			196.6 ± 7.0 mL/kg/km (MAS average = 14.1 km/h)	No training performed	195.2 ± 7.2 mL/kg/km (MAS average = 14.1 km/h) 0.71 ± 0.29%↑
Grieco, et al. [32]	15 Division 1A female soccer players (19.0 ± 0.7 years)	Running on a treadmill at 9 km/h for 5 min; then speed increased 1 km/h every 2 min, until the subject was unable to maintain the pace. Movement economy was calculated for the first 5 min run at 9 km/h.	Off-season	Data not shown	11 weeks off-season combined lower body resistance-plyometric training with a 7-day break during the 7th week. Resistance training 2 days per week on non-consecutive days. 60 min session Consisting of 9–10 exercises of 3 sets each. Plyometric raining conducted on different non-consecutive days. 60 min during the first 4 weeks and approximately 30 min during the final 6 weeks.	Data not shown
						No significant changes reported
Gunnarsson, et al. [35]	7 male soccer players from a team in the Danish Second Division (23.3 ± 0.9 years)	Running for 4 min at a 10 km/h on a treadmill, resting 2 min and then running another 4 min at 14 km/h. Movement economy was calculated both at 10 and 14 km/h.	In-season	35.9 ± 0.9 mL/kg/min (10 km) 47.5 ± 0.7 mL/kg/min (14 km/h)	SET six to nine 30-s intervals per week at an intensity of 90–95% of max intensity, interspersed with 3 min of rest. In the first week of the SET intervention, the players performed five 30-s intervals and one interval was added every week.	33.8 ± 0.9 * mL/kg/min (10 km/h) ↑6% 46.1 ± 1.0 mL/kg/min (14 km/h)

NOTES: HR_max_: maximum heart rate; ME: movement economy; HIIT: high intensity interval training; SET: speed endurance training; SSG: small sided games; vLT_an_: velocity at the anaerobic lactate threshold; MAS: maximal aerobic speed; ↑: improvement; * different between pre- and post-intervention; ** different between study groups.
